# Representations of On-Going Behavior and Future Actions During a Spatial Working Memory Task by a High Firing-Rate Population of Medial Prefrontal Cortex Neurons

**DOI:** 10.3389/fnbeh.2020.00151

**Published:** 2020-08-31

**Authors:** John J. Stout, Amy L. Griffin

**Affiliations:** Griffin Laboratory, Department of Psychological and Brain Sciences, University of Delaware, Newark, DE, United States

**Keywords:** spatial working memory, medial prefrontal cortex, linear classifier, task coding, delayed-nonmatch- to-position

## Abstract

Spatial working memory (SWM) requires the encoding, maintenance, and retrieval of spatially relevant information to guide decision-making. The medial prefrontal cortex (mPFC) has long been implicated in the ability of rodents to perform SWM tasks. While past studies have demonstrated that mPFC ensembles reflect past and future experiences, most findings are derived from tasks that have an experimental overlap between the encoding and retrieval of trajectory specific information. In this study, we recorded single units from the mPFC of rats as they performed a T-maze delayed non-match to position (DNMP) task. This task consists of an encoding dominant sample phase, a memory maintenance delay period, and retrieval dominant choice phase. Using a linear classifier, we investigated whether distinct ensembles collectively reflect various trajectory-dependent experiences. We find that a population of high-firing rate mPFC neurons both predict a future choice and reflect changes in trajectory-dependent behaviors. We then developed a modeling procedure that estimated the number of high and low-firing rate units required to dissociate between various experiences. We find that low firing rate ensembles weakly reflect the direction that rats were forced to turn on the sample phase. This was in contrast to the highly active population that could effectively predict both future decision-making on early stem traversals and trajectory-divergences at T-junction. Finally, we compared the ensemble size necessary to code a forced trajectory to the size required to predict a decision. We provide evidence to suggest that a larger number of highly active neurons are employed during decision-making processes when compared to rewarded forced behaviors. Together, our study provides important insight into how specific ensembles of mPFC units support upcoming choices and ongoing behavior during SWM.

## Introduction

The prefrontal cortex (PFC) is generally defined by anatomical features that include dense mediothalamic afferents and dopaminergic projections from the ventral midbrain (Heidbreder and Groenewegen, 2003). In humans, dorsal-lateral PFC (dlPFC) functionality is abnormal in many neuropsychiatric conditions such as schizophrenia, with corresponding deficits in working memory (Weinberger et al., 1986; Callicott et al., 2000; Manoach et al., 2000; Perlstein et al., 2001; see Manoach, 2003 for a review), the ability to hold information “on-line” (Baddeley, 1992). Like the dlPFC in primates, the rodent medial PFC (mPFC) is important for spatial working memory (SWM; Eichenbaum et al., 1983; Granon et al., 1994; Lee and Kesner, 2003; see Kesner and Churchwell, 2011 for a review; Sapiurka et al., 2016) For over two decades, studies have used *in vivo* recording procedures to better understand the function of the mPFC during memory-guided behaviors (Poucet, 1997; Jung et al., 1998; Baeg et al., 2003; Euston and McNaughton, 2006; Hyman et al., 2012; Yang et al., 2014; Ito et al., 2015; Hallock et al., 2016). However, because few studies have experimentally separated encoding and retrieval processes during SWM in conjunction with mPFC recordings, we lack a complete understanding of prefrontal representations during these distinct SWM components.

Unlike recordings from the dorsal CA1 region of the hippocampus (HPC), where single-units can be modulated by space (i.e., place cells—O’Keefe and Dostrovsky, 1971) or other event-sequences (Pastalkova et al., 2008; MacDonald et al., 2011; Aronov et al., 2017), the mPFC has diverse behavioral-correlates (Jung et al., 1998). Likewise, similar to hippocampal place cells, mPFC neurons exhibit rate modulations that are dependent on the direction by which rats turn at the T-junction (Frank et al., 2000; Wood et al., 2000; Ferbinteanu and Shapiro, 2003; Ito et al., 2015, 2018). Using population decoding techniques, it has been demonstrated that mPFC firing rates can collectively reflect future (prospective) choices as rats approach a decision point on spatial-alternation tasks (Ito et al., 2015; Guise and Shapiro, 2017). However, while *post hoc* analyses can estimate future coding, each traversal of an alternation task contains components of memory encoding and retrieval. Therefore, it is difficult to determine whether firing rate differences truly reflect future trajectories. A task that experimentally separates encoding dominant and retrieval dominant processes could confirm that mPFC ensembles reflect prospective experiences. One such task is the delayed non-match to position (DNMP) task, in which encoding dominant processes occur during the sample phase, and retrieval dominant processes occur during the choice phase.

Another feature of mPFC ensembles that may allow the mPFC to track various features of memory processes during DNMP performance is the diversity of firing rate dynamics within the population. A recent study identified subsets of slow-firing and fast-firing hippocampal units that differed in plasticity and coding properties (Grosmark and Buzsáki, 2016). Additionally, two recent studies focused on sub-populations of rate-modulated mPFC neurons (Spellman et al., 2015; Bolkan et al., 2017), finding evidence that distinct anatomical projections support specific mPFC neuronal activity. Furthermore, Spellman et al. (2015) revealed that the mPFC population could predict whether mice were on the sample or choice phase of a DNMP task, similar to dCA1 neurons in rats (Griffin et al., 2007). Thus, we hypothesized that separate mPFC populations, split according to firing-rate profiles, would differentially represent task features.

To address this hypothesis, we used supervised machine learning trained and tested to predict various classes of interest, specifically task phase (sample or choice), trajectory (left or right), or various combinations of task-phase and trajectory. Given that at least three separate paradigms have been used to demonstrate that single-unit activity in the mPFC is modulated by trajectory-dependent behaviors (Euston and McNaughton, 2006; Ito et al., 2015), we analyzed lateral position changes (detected using an LED on the rats head) in conjunction with neuronal decoding on the stem portion of the DNMP task. Here, we show that a sub-population of fast-firing neurons strongly reflects trajectory-dependent behaviors (whether rats running direction diverges towards the left or right reward-well, at the T-junction) and future decisions. We also characterize the low-firing rate populations by developing a simple modeling procedure. This approach revealed that low-firing neurons weakly represent a forced trajectory-dependent behavior (whether rats turned left or right at T-junction during sample traversals), but not future decisions, suggesting a conserved role for representing actions across mPFC populations. Finally, in the development of the modeling procedure, we discovered that larger ensembles are required to predict a future decision when compared to a forced trajectory that always results in a reward. Together, this study provides important extensions to our understanding of how the mPFC contributes to SWM.

## Materials and Methods

### Subjects

Subjects were five adults (>90 days), male, Long-Evans hooded rats purchased from Harlan, Laboratories. During the experiment, rats were put on mild food restriction to maintain them at ~90% of their *ad libitum* body weight. The colony room was humidity and temperature-controlled with a 12-h light/dark cycle. All experiments were performed during the light cycle.

### Behavioral Apparatus

**Figure 1:**
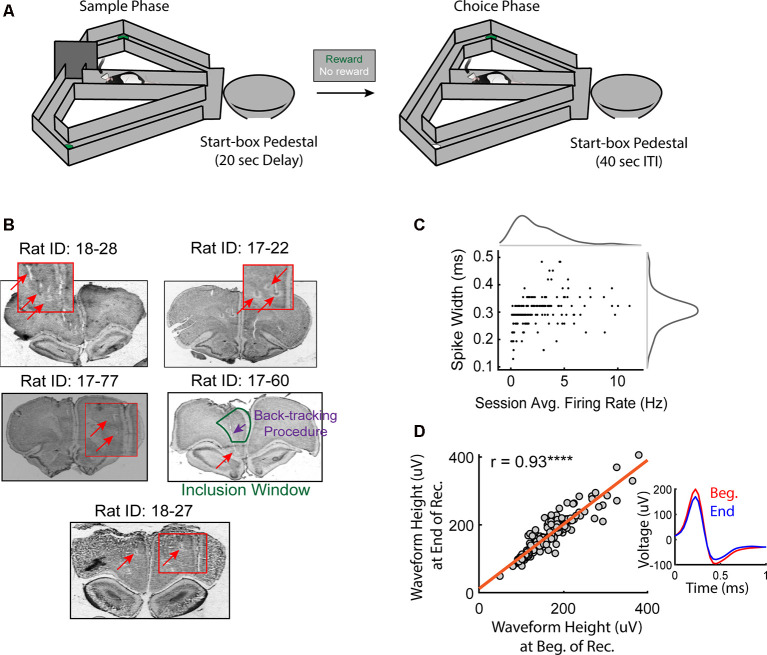
Histological confirmations and recording stability. **(A)** Schematic of the delayed non-match to position (DNMP) task. Each trial of this task includes two traversals that are separated by a 20 s delay: a sample traversal in which one of the goal arms is blocked, forcing the rat to choose to enter the opposite goal arm and a choice traversal in which the rat is free to choose a goal arm but only rewarded for choosing the goal arm opposite to the arm visited on the sample traversal. Trials are separated by a 40 s inter-trial interval (ITI). For a more detailed description of the DNMP task, see “Materials and Methods” section. **(B)** Histological confirmations of final tetrode locations in the medial prefrontal cortex (mPFC). Note that in some cases, the final locations of tetrodes exceeded the window of inclusion (prelimbic and anterior cingulate cortex). Tetrode locations on specific recording days were estimated from the turn-count and final location of the tetrode (see “Materials and Methods” section). Red boxes are magnified images of the tetrode lesion (halo-marking) and/or the tetrode track (space). **(C)** Spike-widths were plotted against the session averaged firing rates for all included pyramidal neurons. Notice the Gaussian-like distribution for spike-widths in comparison to the session-averaged firing rates. **(D)** Only stable clusters were included for analysis. The average peak waveform height during the first 10 min of recording was strongly correlated to that of the last 10 min (*r* = 0.93, ****p = 1.6e-81). The inset shows the average waveform of a representative neuron during the first (red) and last (blue) 10 min of the recording session.

The behavioral apparatus was an elevated, wooden T-maze, painted black with a stem (127 × 9 cm), two goal-arms (50 × 9 cm), two goal-zones (37 × 9 cm), two return-arms (130 × 9 cm), and 5.6-cm high wooden walls. The start box, where rats were confined during the delay and inter-trial interval (ITI) periods, was a plastic bowl attached to a bar stool that was located at the base of the T-maze stem (see Figure 1A). The right and left goal zones were located at the end of each goal arm and contained a small plastic dish in which the chocolate sprinkle reward was placed. The maze was surrounded by black-curtains with large visual cues (made using colored tape). The testing room was dimly illuminated with one compact fluorescent bulb.

### Handling and Pre-training

Handling and pretraining methods were similar to those used previously in our laboratory (Hallock et al., 2013a; Hallock and Griffin, 2013b). Briefly, rats were handled for 10–15 min a day for 5 days before the experiment began. After each handling session, chocolate sprinkles to be used for a food reward in the task were placed in a small dish in the home cage. Pre-training consisted of “reward-zone” habituation sessions, followed by “forced run” behavioral shaping sessions. During each reward zone training trial, rats were confined to one of the two-goal zones and given 3 min to eat the chocolate sprinkle reward; animals only progressed to the next pre-training procedure if they consumed the reward within 90 s on all trials. Each reward zone training session consisted of six-goal zone visits, alternating between the right and left goal zone. Once rats consumed the reward on every trial for two consecutive goal zone training sessions in under 90 s, they were advanced to forced run training, which consisted of 12 trials per session. During each forced run training trial, one of the goal arms was pseudo-randomly selected (Fellows, 1967) to be blocked off with a tall wooden barrier. Rats were placed in the start box and allowed to run up the maze stem and into the open goal arm, where they received a reward. They then returned to the start box *via* the return arm. Once rats ran on the maze without turning around and consumed the food reward on all forced-run trials for at least one session, they proceeded to DNMP training. A schematic of the DNMP task can be found in Figure 1A. Each trial consisted of a sample traversal, a delay period, and a choice traversal. Before each trial, while rats were confined to the start box by a barrier, the experimenter baited both reward zones with chocolate sprinkles and placed the goal arm barrier at the entrance of either the right or left goal arm. To initiate a trial, the experimenter lifted the start box barrier, allowing the rat to run up the stem and turn into the open goal arm. After consuming the reward, the rats returned to the start box *via* the return arm and were held in the start box for a 20 s delay period. After the delay period, the experimenter removed the start box barrier to allow free access to the stem. The rats again traversed the stem but this time were free to turn into the left or right goal arm. Only the reward zone opposite to the reward zone visited on the sample traversal was now baited (green color in depiction) so that rats were rewarded for implementing an alternation rule. Trials were separated by a 40 s inter-trial interval (ITI). Left and right sample traversals were given in a pseudo-randomized sequence, with no more than three turn directions given consecutively and equal numbers of left and right trials given within a session.

### Surgery Protocol

Once the DNMP criterion was met, rats were implanted with a micro-drive consisting of 8 or 14 independently moveable tetrodes, made from four 13.5 μM nichrome wires, twisted together (Kanthal Palm Coast) targeted at the mPFC (*N* = 5 rats). Some rats were implanted with electrodes in the dorsal hippocampus and/or ventral midline thalamus along with virus injection for optogenetic manipulations. These data were used for a separate experiment.

Before incision, the surgeon ensured that the rat was fully anesthetized by checking for a leg reflex following a foot pinch. Before the incision was made, lidocaine was injected subcutaneously at the incision site. A craniotomy was made at 4.2 mm posterior and 2.4 mm lateral to bregma for hippocampal LFP wires, and 2.3 mm posterior and 2 mm lateral at a 15° angle to bregma for nucleus reuniens LFP wires. For the mPFC micro-drive, a craniotomy was made at 3.25 mm anterior and 1.15 mm lateral to bregma and implanted on a 7° angle (all recordings were done on the left hemisphere mPFC). The implant was anchored to the skull in order from caudal-to-rostral implants to a total of seven bone screws (six small bone-screws and a larger bone-screw for grounding; Fine Science Tools) *via* acrylic (Lang Dental). Toward the last 20 min of surgery, rats were injected with Banamine and given Children’s Ibuprofen in their water for ~5 days post-surgery. Rats were administered prophylactic antibiotics for 1-week post-surgery and no recordings were included from this period. All performed procedures are recognized and approved by the University of Delaware Institutional Animal Care and Use Committee (IACUC).

### Single-Unit Recordings

Rats were allowed to recover for 5 days in their home cages. They were then re-handled and habituated to the recording room by sitting in a large plastic enclosure while receiving Kellogg’s Froot Loops. Before each recording session, tetrodes were advanced into the brain and allowed to settle for 30–60 min. Digital Lynx (Neuralynx) 64-channel recording system was utilized for data acquisition. For video-tracking, a camera was mounted on the ceiling, and rat position was acquired *via* LED’s that were attached to the rat’s micro-drive for recording. Position and time-stamps were acquired at a 30 Hz sampling rate. Single-cell spiking was sampled at 32 kHz and band-pass filtered between 0.6–6 kHz, with thresholds being set between 50 and 75 μV. References for tetrode recordings varied depending on the session. Rats ran at least 18 trials each day. If a session exceeded 18 trials, only the first 18 were included in the analysis.

### Perfusion and Histology

After the final recording session, electrolytic lesions were created by passing ~11.9 μA of current through each wire for all tetrodes including the reference tetrodes, using the Neuralynx nanoZ kit. Lesions were allowed to develop between 1 and 3 days before rats were anesthetized, and a lethal injection of sodium pentobarbital was given. Following the perfusion, the head was soaked in 4% PFA or formalin for 1–3 days. Once the tetrodes were removed from the brain, the brain was extracted and placed into a 30% sucrose solution. After sinking, brains were frozen and sectioned in the coronal plane using a cryostat and mounted on slides. To identify tetrode lesions and tracks, a Cresyl Violet stain was used for imaging with the Northern Lights Imaging Illuminator.

### Estimation of Electrode Depth

To estimate the tetrode location, we utilized a known conversion between turn-count and millimeters. We localized tetrodes based on a variety of factors including a mapped bundle of all tetrodes, the bundle width, bundle length, tetrode depth, and surrounding tetrode tracks/lesions. If tetrode tracks were not immediately apparent, we utilized the center point of the lesion based on dual identification of lesions and tetrode tracks (Figure 1B) to estimate the tetrode’s final location. If we noticed a rat with a lesion and tetrode end-mark that was slightly lower or higher than the center-point of the lesion, we used that point as the final tetrode location for the rest of the tetrodes for that rat. Nonetheless, all tetrode final locations were very close to the center-point of the lesion. Once tetrodes were localized, we estimated previous session tetrode depths,

$$\text{Depth = [Final turncount (in mm)}-{\text{Session}}_{i}\text{ turncount (in mm)}]+\text{Final DV coordinate (in mm) of the tetrode in the brain}$$

where *i* corresponds to a previous session of interest. This allowed us to use the difference between turn-counts (in mm), add them to the final localized tetrode DV coordinate to gain an estimate of the previous location of the tetrode using the Paxinos and Watson brain map atlas (Paxinos and Watson, 2006).

### Cell Isolation and Stability Evaluation Procedures

Clusters of spikes were identified visually using SpikeSort 3D (Neuralynx), cut automatically using Klustakwik, and manually sorted/combined based on less than 25% visually identified overlap with noise, spike waveform height, and peak-amplitude. L-ratios were calculated for each cluster using David A. Redish’s MClust MATLAB package and only included if the value was less than 0.1 (Schmitzer-Torbert et al., 2005). Putative pyramidal cells were identified based on interspike-intervals and spike waveform shape (Ranck, 1973; Figure 1C). Only clusters that were stable across the recording session were included for further analysis (Figure 1D). To assess cluster stability, for each cluster, we first used SpikeSort3D to visualize changes in waveform peaks across the recording session and excluded any clusters that showed a marked change in waveform peak across the session. To demonstrate the validity of this approach, for each of the remaining clusters, we selected the channel with the highest session-averaged peak amplitude and compared the mean peak amplitude (“waveform height”) for spikes recorded during the first 10 min to the mean peak amplitude for spikes recorded during the last 10 min using the Pearson correlation coefficient.

### Linear Classification

To assess the degree by which the population of mPFC neurons collectively represented different behavioral classes, we used a binary support vector machine (SVM) linear classifier from the LIBSVM toolbox (Chang and Lin, 2011). During the training phase, an SVM linear classifier generates a linear line (hyperplane) in a mapped high-dimensional space of firing rate vectors that separate user-defined class. The hyperplane is selected based on the margin that maximally separates the nearest mapped firing rate vectors (support vectors) in the high dimensional space (Müller and Guido, 2016). During the testing phase, the classifier assigns the firing rate vector as belonging to one of two classes, defined by an integer [i.e., sample (1) vs. choice (−1)]. These integers are further referred to as “labels”. If the classifier is correct, the output variable is a 1, if incorrect, the output variable is a 0. The regularization parameter C was set to 1, similar to a past study from our lab (Hallock et al., 2016). Our classification procedures utilized a leave-1-out approach, where one population vector was removed from the training dataset to act as the testing vector. The classifier was trained/tested on every trial, one time, using this method. More specific mathematical descriptions of how the classifier accomplishes the training and testing of vector-formatted data can be found elsewhere (Chang and Lin, 2011; Hallock et al., 2016).

This study utilized a pseudo-simultaneous approach (Spellman et al., 2015) whereby a single population vector contained firing-rates from all recorded neurons across sessions and rats. In other words, per the training and testing dataset, there was an N (trials) × M (neurons) matrix of firing rates. The pseudo-simultaneous method was chosen since preliminary analyses revealed a significant positive relationship between the number of added neurons per session and classifier performance (*R* = 0.6744, *p* = 3.78e-07), making it difficult to interpret session-averaged accuracies using data collected from this study.

**Figure 2:**
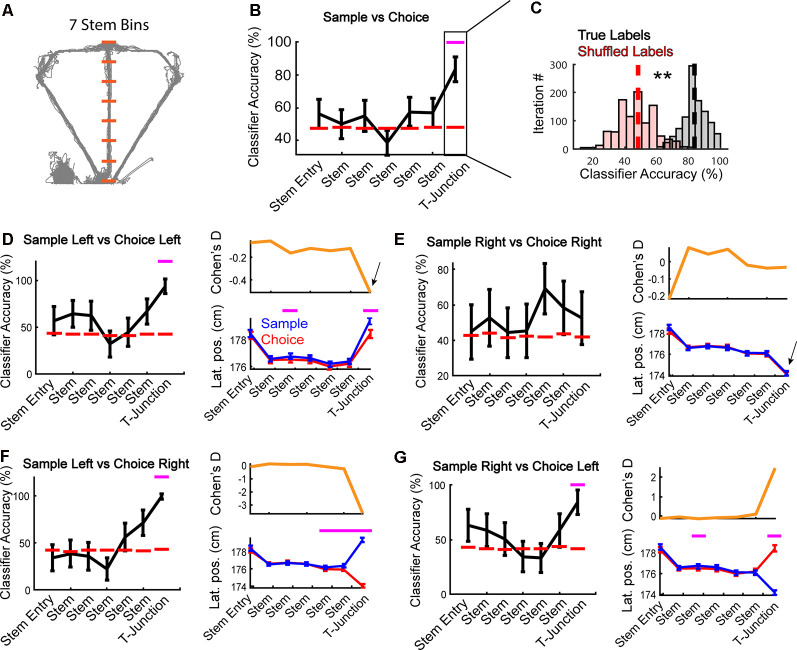
Apparent encoding of task phase is supported by representations of trajectory-dependent behaviors. **(A)** Depiction of stem bins superimposed on position data from an example session (space between orange dashes) used in the following analyses. Orange lines highlight the start/endpoints for spatial bins on the maze used in panel **(B)**. The 7th bin from the start of the stem was defined as a T-junction (see “Results” section). **(B)** A linear classifier was trained to predict the task phase (sample vs. choice) across all stem bins (*N* = 187 neurons per bin). Notice that task phase is accurately predicted at T-junction (7th bin/T-junction: *Z* = 3.1, *p* = 0.002 *z*-test against shuffled distribution). Data are represented as mean ± standard deviation of 1,000 classifications. Magenta dashes indicate a *p*-value <0.05. **(C)** A demonstration from the T-junction of how our classification averages were obtained (see “Materials and Methods” section). A *z*-test was then performed using the mean classification accuracy on true labels, against a shuffled distribution. Dashed lines indicate the average classification accuracy across iterations. **Indicates *p* < 0.01. Statistics are identical to that in panel **(B)**. **(D)** Left panel: the mPFC was trained to predict whether rats were on a sample or choice phase during left trajectory runs. The classifier could accurately predict task phase at T-junction (*Z* = 2.9, *p* = 0.004). Right panel: session averaged lateral position (“Lat. pos”) data revealed that rats lateral position at early stem and T-junction varied with task phase (main effect of task phase *F*_(1,6)_ = 4.75, *p* = 0.03; bin 3: *t*_(43)_ = −3.5, *p* = 0.001; bin 7: *t*_(43)_ = −5.1, *p* = 7.8e-06). **(E)** The mPFC was trained to predict task phase from right trajectory runs. Notice that at T-junction, the classifier did not significantly predict task phase (*Z* = 0.57, *p* = 0.572). Right panel: there was no main effect of task phase (*F*_(1,6)_ = 0.08, *p* = 0.7), or task phase × bin interaction (*F*_(1,6)_ = 0.31, *p* = 0.9). **(F)** A right turn on choice phase always followed a left turn on the sample phase in the included data (only correct trials were included). The mPFC only predicts left samples/right choices at T-junction (*Z* = 3.0, *p* = 0.002), the location where lateral position (right panel) differs. There was an interaction between task phase and bin (*F*_(1,6)_ = 41.98, *p* = 5.7e-43). Paired *t*-tests revealed significant difference in lateral position based on task phase—trajectory combination (bin 5: *t*_(44)_ = −3.1, *p* = 0.003, bin 6: *t*_(44)_ = −4.2, *p* = 0.0001, bin 7: *t*_(44)_ = −36.9, *p* = 9.3e-35). **(G)** Choice lefts always followed sample rights in the included data (correct trials). We found that sample right and choice left neuronal activity was functionally distinct at T-junction (*Z* = 2.5, *p* = 0.014). This effect coincided with changes in the rat’s lateral position (*F*_(6)_ = 26.1, *p* = 1.22e-27 interaction between the task phase and bin). Classification data are represented as mean ± standard deviation with magenta lines indicating *p* < 0.05 from a *z*-test. Behavioral data are represented as mean ± SEM with magenta lines indicating *p* < 0.007 from a *t*-test with a Bonferroni corrected alpha of 0.007.

To control for any effects of matrix organization on classification accuracy, we iteratively selected N-number of random trials per neuron within a given class, 1,000 times. In other words, per iteration (1,000 per stem bin), there was a different training/testing dataset that retained class while controlling for matrix organization (i.e., row 1 is not always trial 1 for every neuron). We refer to this method as a “permutation-style” classification approach. To train/test the classifier on correct trials, we found the session that had the least number of correct trials (15 trials for task phase, six trials for any other class), then iteratively selected N-number of random trials for each neuron, 1,000 times. This created 1,000 iterations of random combinations of trials for each neuron, thereby allowing us to use only correct trials while also controlling for any chance that our classification results were obtained by an ordering effect of trials. Therefore, for any analyses where we used this method (all except Figures 4A through 4E), the data are displayed as the average of 1,000 different classification accuracies that retain class while controlling for matrix organization (see Figure 2C for an example distribution). Code is available and can be found on the lab’s GitHub^1^ webpage.

**Figure 3:**
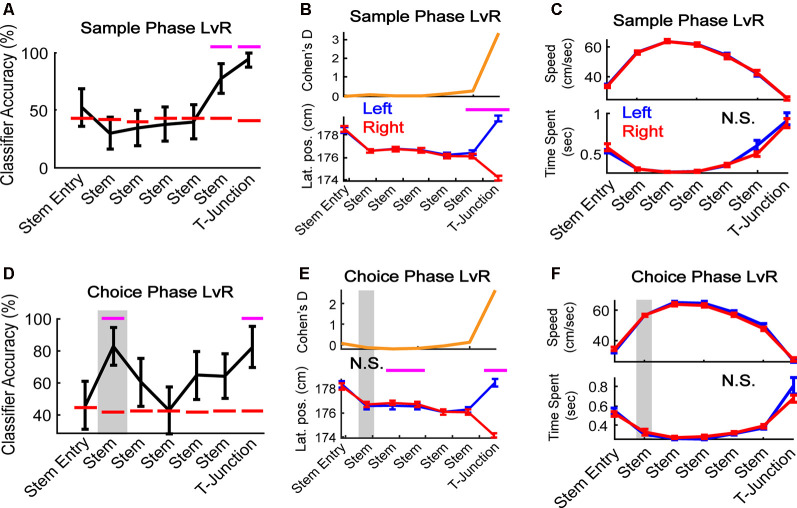
mPFC population activity predicts a future decision. **(A)** During sample traversals, the mPFC population predicted trajectory (left vs. right turns) as rats approached and occupied T-junction (bin 6: *Z* = 2.0, *p* = 0.049; bin 7: *Z* = 2.9, *p* = 0.004). *N* = 187 neurons, six random trials were selected per class and neuron (see “Materials and Methods” section), 1,000 times. **(B)** Rats altered their lateral positions in a manner consistent with classification in panel **(A)**. There was a significant interaction between task phase and bin (*F*_(1,6)_ = 39.63, *p* = 1.3e-40). Bins 6 and 7 survived Bonferroni correction (*t*_(43)_ = 3.4, *p* = 0.001; *t*_(43)_ = 31.3, *p* = 3.3e-31; Bonferroni corrected alpha = 0.007). *N* = 44 sessions. **(C)** Differences in time-spent at the T-junction and velocity do not account for the mPFC predicting the future trajectory. There was no main effect of trajectory (*F*_(1,6)_ = 0.08, *p* = 0.77) or interaction between sample left/right velocity and bin-location (*F*_(1,6)_ = 0.05, *p* = 0.99). N.S. indicates no significance. **(D)** During choice phase, the mPFC predicted the future (Stem bin 2: *Z* = 2.28, *p* = 0.023) and current (T-junction: *Z* = 2.2, *p* = 0.028) trajectory. For classification procedures: *N* = 187 neurons, six random trials were selected per class and neuron, 1,000 times. Magenta lines indicate *p* < 0.05 using a *z*-test against a shuffled distribution. Gray box highlights the second bin where the mPFC could predict the future left or right, and is used in the following panels. Data are represented as the mean ± standard deviation. **(E)** Lateral positions do not change in the stem location where the population predicts a future decision in panel **(D)**. There was a significant interaction between choice trajectory and stem bin (*F*_(1,6)_ = 27.6, *p* = 3.8e-29). Stem bins 3 and 4, and T-junction, survived Bonferroni correction (*t*_(43)_ = −4.3, *p* = 0.0001, *t*_(43)_ = −3.5, *p* = 0.001, *t*_(43)_ = 24.1, *p* = 1.2e-26; Bonferroni corrected alpha = 0.007). **(F)** There was no main effect of task phase nor interaction for choice left vs. choice right for velocity (top panel *F*_(1,6)_ = 1.25, *p* = 0.26; *F*_(1,6)_ = 0.97, *p* = 0.45) or time spent (bottom panel *F*_(1,6)_ = 0.45, *p* = 0.5; *F*_(1,6)_ = 1.86, *p* = 0.085. Gray box highlights the bin where classification accuracy was above chance. For behavioral analyses, data are represented as the mean ± SEM. Paired *t*-tests were performed, but only those that survived Bonferroni correction were plotted with magenta lines. N.S., not significant.

**Figure 4:**
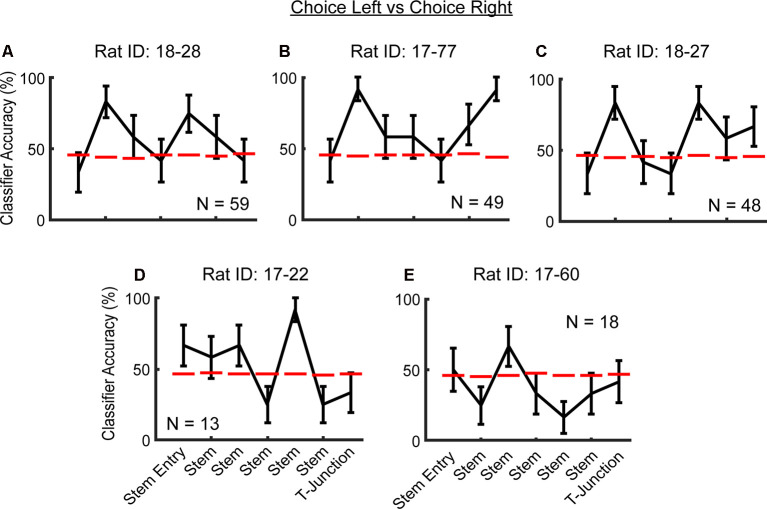
Consistency in future action prediction of mPFC ensembles across rats. **(A–C)** Notice the peak in classification accuracy in the second stem bin. Additionally, note that in rats **(A–D)**, there are peaks in classification accuracy that are well above chance threshold, before T-junction entry. Data are displayed as the mean ± standard deviation of classification accuracies for all trials. The dashed red line indicates a chance level distribution generated using 1,000 shuffled labels. Dashed magenta lines indicate a significant effect that does not survive a corrected alpha. **(D–E)** Rats with lower sample sizes of recorded neurons did not exhibit the effect at the second stem bin.

### Modeling Classification Accuracies

Linear classifier accuracies and their corresponding sample sizes (number of added neurons) were log-transformed and multiplied by 10. Linear regression was performed on the two log-corrected vectors using MATLAB’s fit and fitlm functions. After obtaining the model formula, and calculating the log-transformed prediction accuracies, we re-converted the data to classification accuracies using the anti-log. Significance was estimated by comparing the predicted accuracy estimates to a shuffled distribution using a *Z*-test (see “Statistical Procedures” section). The code can be found in the same location as mentioned above.

### Statistical Procedures

To determine if classification accuracies were at a level above chance, labels (1’s or −1’s—see “Materials and Methods” section on linear classifier) were shuffled and the classifier was trained/tested 1,000 separate times using data derived from the final iteration (out of 1,000) of the true labeled data. This approach created a normal distribution to estimate chance level performance. The mean and standard deviation of a shuffled distribution (1,000 shuffles) was obtained and compared against the mean from the 1,000 iterations of the true labeled data using a *z*-test (see Sangiamo et al., 2020). Corrected alphas were not utilized on permutation-style classifications for two reasons: (1) these analyses were planned comparisons, whereby we only compared iterative classification averages to a shuffled average; and (2) we utilize an iterative approach (1,000–5,000 independent random trial shuffles per neuron while retaining class, depending on the analysis) which controls for any spurious effects driven by trial order.

To assess behavioral trajectory changes between task phases or trajectories, and to assess whether firing rates were dependent on turn direction, two-way ANOVAs were used with one factor being a stem bin and the other being class (task phase—sample/choice or turn direction—left/right). When follow-up paired *t*-tests were performed (only between classes at each stem bin), a Bonferroni corrected alpha was used to assess significance. Cohen’s *d* was used to quantify the effect size in certain analyses. Pearson’s correlation was used to assess relationships between two variables when appropriate.

## Results

### Apparent Task Phase Decoding Is Better Explained by Representations of Trajectory-Dependent Behaviors

To assess if task coding (i.e., the ability to distinguish between encoding dominant sample phases and retrieval dominant choice phases) was present among the collective population of mPFC units, we first determined that seven spatial bins isolated the T-junction from the rest of the stem (Figure 2A). We operationally defined the T-junction as the physical space where the rat initially encountered the sample phase barrier and includes a decision (a turn onto the goal-arms). Given that the final bin was similar in area to our operational definition of T-junction, we used this number of bins for the remainder of our analyses. Other studies have used similar binning of the T-junction (Wood et al., 2000; Yamamoto et al., 2014; Hallock et al., 2016).

First, we examined if the mPFC could predict the task phase, similar to what has been observed previously in mice (Spellman et al., 2015). In contrast to that study, where the mPFC predicts task phase upon stem entry, we found that mPFC ensembles maximally reflect the task phase at T-junction (Figures 2B,C). We then examined whether the ability to predict the task phase was trajectory-dependent. To do so, we trained the classifier to predict sample and choice phases on the left and right trials, separately. Surprisingly, task phase coding was robust on left, but not right traversals (Figures 2D,E left panels). This effect mirrored changes in lateral positions, such that lateral position only significantly differed on left, but not right traversals (Figures 2D,E right panels). We then examined individual rat behavior and observed that two rats showed no significant change in a lateral position on left trials. Nonetheless, their exclusion did not alter classification accuracy (data not shown). In contrast to left runs, no rats exhibited a significant change in lateral position between task phases on the right trajectories (data not shown). Therefore, the lateral position, not the context of the task phase, seems to drive mPFC predictions.

Next, we compared task-phase trajectories that were always paired during correct decision-making (i.e., a left choice always followed the right sample). If the mPFC represented information linked to tracking the rule associated with the task phase and trajectory, then we would observe high classification accuracy in the absence of behavioral differences. On the contrary, if we only observe the population to predict the task-phase trajectory combination at T-junction (where lateral position divergences maximally), then it would support the notion that the mPFC primarily tracks trajectory-dependent behaviors. Consistent with Figure 2D, we only found the classifier to perform above the chance level when lateral positions differed at T-junction (Figures 2F,G). Together, these findings reveal that changes in lateral positions are the most parsimonious explanation for apparent task phase coding among the population of mPFC neurons. These results are also in-line with past reports that revealed strong mPFC representations linked to trajectory-dependent head-movements (Euston and McNaughton, 2006; Ito et al., 2015).

### The mPFC Reflects Overt Action and Planned Behavior

Given that our findings shown in Figure 2 points towards the mPFC playing a dominant role in tracking trajectory-dependent behaviors, we wondered if the mPFC represented distinct trajectories within the sample and choice phases. Because the sample phase trajectory is pseudo-randomized, rats should not be able to predict whether their future direction is left or right until they approach the T-junction. However, if the mPFC were to reflect planned action, then we should find the population to reflect a future decision with minimal changes in lateral position. To address these ideas, we trained the classifier to predict left vs. right turns on the sample and choice phases separately (Figures 3A,D). During the sample phase, the mPFC population predicted the current trajectory as rats approached and occupied the T-junction (Figure 3A). This finding mirrored reliable changes in lateral positions across sessions (Figure 3B) but did not reflect changes in speed nor time-spent at the T junction (Figure 3C). However, consistent with the idea that the mPFC reflects planning of future trajectories during stem running (i.e., prospective coding—Ito et al., 2015), we found that the mPFC population predicted the future decision during early stem traversal (Figure 3D, the second stem bin is the first location where the rat’s body is completely enclosed by maze walls—see Figure 1A). This effect occurred in the absence of differences between lateral position, running speed, and time spent in the second stem bin (Figures 3E,F).

Next, we wondered if our effects were present when using a simpler classification method whereby each row is always the same trial per neuron (the temporal organization of the data is not being controlled). This approach is commonly used (Ito et al., 2015; Hallock et al., 2016). Therefore, we trained/tested the classifier to predict left and right turns on the choice phase using the first six left trials and first six right trials (note that the shape of the training/testing matrix has to be symmetrical, and six trials were the smallest number of left and right traversals across sessions). To account for any effects of multiple comparisons, we used a Bonferroni corrected alpha level of 0.0071. Pre-alpha correction, we found the classifier to perform at 100% in the second stem bin, and 83% in the fourth– T-junction bins. After alpha correction, the effect at the second stem bin survived (*Z* = 2.95, *p* = 0.0032; data not shown). Thus, our permutation-style classification methodology was more conservative than the classical approach and accounted for potential model overfitting (classifier accuracy performing at 100%) by breaking the temporal organization of the data (Figure 3D).

To then determine whether the ability to predict a future trajectory during the choice phase was consistent across rats, we pooled neuronal data across sessions, but within rats (within-rat pseudo-simultaneous approach). Given the comparably smaller sample sizes of neurons per rat when compared to the total population (Figures 4A through 4E), we again used a less conservative classification approach in which we trained/tested the classifier on the first six left choice trials and the first six right choice trials, one time per rat. We found that three out of five rats (those with >40 units) exhibited peaks in classification accuracy of the future trajectory during the choice phase, in the second stem bin (Figures 4A through 4E). The exact location along the end of the choice traversal where mPFC ensembles predicted the upcoming choice was variable across rats. In two out of five rats, there were peaks in classification accuracy in the fifth stem bin (Figures 4C,D), whereas, in one rat, mPFC ensembles predicted the upcoming choice at the T-junction (Figure 4B). It should be noted that in four of five rats, the classifier performed well above the chance threshold before T-junction occupancy (Figures 4A–E). We suspect that the variability in the location of ensemble trajectory coding could be due to variability in what point along the trajectory the rats make their decision. Alternatively, this variability may be due to poor sampling of the T-junction by the recorded neurons in some rats. Nonetheless, the mPFC unit population is consistently able to predict a future decision.

Finally, we wondered if there were a large proportion of instances whereby the classifier performed above chance level in Figure 3D. Therefore, per classification iteration (1,000 in total), we used a *z*-test to determine if the model performance was above the mean and standard deviation of the shuffled distribution. First, we performed the *z*-testing procedure on all instances of classification outcomes from stem bin 1 (“Stem Entry”), as this stem bin performed near chance level. We predicted that about 5% of instances would be significant by chance alone; however, we found that 2% of classifier outcomes performed above chance level, highlighting the conservative nature of our method (data not shown). Then, we examined the proportion of classification accuracies at stem bin 2. As opposed to the 2% of classifier outcomes in a chance level performing classifier, we found that 64% of classifications (where we break the temporal organization of the data while retaining class) were significantly (*p* < 0.05) above chance level (data not shown). Thus, this effect is largely independent of the temporal ordering of trials.

Taken together, we took multiple approaches to demonstrate that the mPFC population robustly predicts a future decision on a DNMP task. These findings suggest that mPFC prospective trajectory coding on the choice phase, occurs before T-junction occupancy, enabling the rat to plan a future decision. Our results also support the mPFC tracking trajectory-dependent behaviors at the T-junction (Figures 2, 3).

### High-Rate Neurons Reflect Current Trajectory Behavior and Predict a Future Decision

As rats explore a novel environment, hippocampal neurons initially classified as having a low firing rate (a firing rate less than the 50th percentile of all neuronal activity during sleep) acquire stronger spatial modulation and exhibit evidence of plasticity during sharp-wave ripple events; this is unlike neurons initially classified with a high-rate profile (Grosmark and Buzsáki, 2016). These findings may be in-line with some place-cells acquiring rate-dependent plasticity, increasing their firing for a specific trajectory (i.e., “splitter cells”) following the learning of a spatial strategy (Dudchenko and Wood, 2014). Therefore, high-rate and low-rate neurons may differentially represent a learned task. However, no study to our knowledge has investigated if high-rate and low-rate neurons in the mPFC better represent features of SWM.

**Figure 5:**
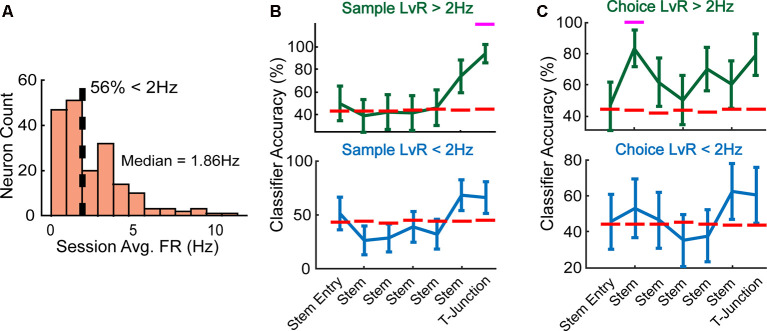
High-rate neurons reflect current and future behavior during well-learned DNMP performance. **(A)** Fifty-six percentage of neurons exhibited a session averaged firing rate <2 Hz, a firing rate that approximated the median of 1.86 Hz. We split the data according to a 2 Hz threshold (dashed line) and define high-rate neurons as having a firing rate that exceeds the 2 Hz threshold, and low-rate neurons as having a firing rate that is below 2 Hz. **(B)** Top panel: high-rate neurons collectively predicted current trajectory at T-junction during sample phase (*Z* = 2.7, *p* = 0.007, *N* = 89 neurons). Bottom panel: low-rate neurons do not collectively reflect trajectory information (*N* = 98 neurons). Magenta lines indicate significance using a *z*-test (see “Materials and Methods” section). Data are represented as mean ± standard deviation of classification accuracies. **(C)** Top panel: high-rate neurons collectively predicted the future trajectory during early stem traversals (*Z* = 2.1, *p* = 0.036), but exhibited a trending effect at T-junction (*Z* = 1.9, *p* = 0.053). Bottom panel: low-rate neurons do not collectively reflect trajectory information on the choice phase. Data are represented identically as described in panel **(B)**.

To split our recorded population into high-rate and low-rate groups, we visualized the mean firing rates across all neurons using a histogram. Upon initial observation, we observed that about 56% of the neurons exhibited a session averaged firing rate of less than 2 Hz (Figure 5A), with 2 Hz being rather consistent with a median split of 1.86 Hz. Thus, we grouped neurons into a high-rate category if they exceeded 2 Hz, and low-rate category if they exhibited a rate less than 2 Hz. When we trained/tested a linear classifier using the high-rate neuronal population, we found that it could accurately decode whether rats were turning right/left on the sample phase (Figure 5B, top panel) and also predict a future decision during the choice phase (Figure 5C, top panel). Interestingly, this was in contrary to the low-rate population, as these neurons did not collectively discern current (Figure 5B, bottom panel) nor future behaviors (Figure 5C, bottom panel).

However, we possibly did not observe any effect in the low-rate population due to lower statistical power compared to the high-rate population. It is well known that SVM based classification methods are sensitive to large scaling differences in the dataset (Müller and Guido, 2016). Therefore, to account for the lower firing-rate values present in the training/testing data of the low-rate group, we scaled firing-rates to range between 0 and 1, per neuron for all iterations. We found no drastic differences in classification performance after scaling the data, for either the high-rate or low-rate groups (data not shown). Thus, scaling differences between features is not a likely explanation for our results. However, it is still possible that low-rate neurons are contributing important information that our methods are unable to detect.

**Figure 6:**
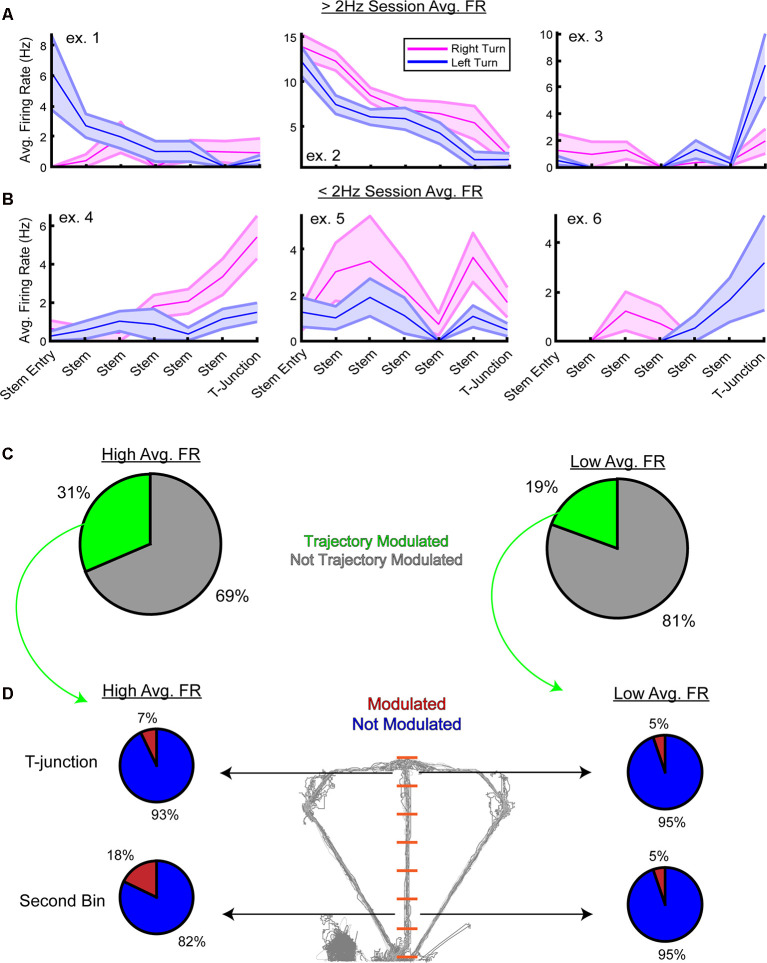
Trajectory modulation is evident at the single-unit level from the high and low-rate populations. **(A)** Three example units from the high-rate population that were significantly modulated by trajectory on the stem of the DNMP task. Using a two-way analysis of variance, a unit was considered “significantly modulated” if there was a significant main effect of trajectory or a significant interaction between stem bin and trajectory on firing rates. Data are displayed as the trial-averaged mean ± SEM of firing rates. **(B)** Three example units from the low-rate population that were significantly modulated by trajectory. Note that while units exhibited a session average of <2 Hz, their firing rates on the stem can be above 2 Hz. **(C)** Pie charts from the high-rate population (left panel) and low-rate population (right panel) demonstrating the proportion of units modulated by trajectory. Note the greater percentage of high-rate neurons that are trajectory-modulated. **(D)** Follow up paired *t*-tests were performed on firing rates from the T-junction and the second stem bin. Alpha level was corrected using Bonferroni’s method (alpha = 0.025) and only units that had a p-value less than the corrected alpha level were included. Notice in the high-rate population, 18% of units were significantly modulated during early stem running, and 7% were modulated at T-junction. This is in contrast to the low-rate population where only 5% of the trajectory-modulated units exhibited preferential firing towards one trajectory at the second stem bin and T-junction. Note that the second stem bin is the first bin where the rat is completely enclosed by stem walls.

Next, we inspected the firing rate preferences of the high- and low-rate populations at the single-unit level. Using a two-way analysis of variance with the firing rate as the dependent variable, a unit was considered trajectory modulated if there was a significant main effect of trajectory or interaction between stem bin and trajectory. Focusing on the choice phase, we first visualized examples of units that were significantly modulated by trajectory (Figures 6A,B). Then, concerning the low-rate population, we predicted the following: (1) there would be a greater proportion of high-rate neurons modulated by trajectory; and (2) a greater number of high-rate neurons would dissociate left/right turns at the second stem bin. As expected, a greater proportion of high-rate neurons were significantly modulated by trajectory (Figure 6C, 31% high-rate neurons modulated, 19% low-rate neurons modulated). To then test if more high-rate units were modulated by trajectory during early stem running and T-junction occupancy, we performed paired *t*-tests on data taken from the second stem bin and T-junction locations. After Bonferroni correction (alpha = 0.025), 18% of high-rate neurons were modulated by trajectory at the second stem bin, while only 5% of low-rate neurons significantly differed in firing rates (Figure 6D). Interestingly, a similar proportion of high-rate units were significantly modulated at the T-junction (7%) in comparison to the low-rate population (5%). Taken together, these results suggest that highly active mPFC populations in well-trained animals collectively represent both current actions and future decisions. Additionally, the ability to dissociate between two possible future actions was apparent at the single-unit and population level.

### A Large Proportion of Neurons Are Required to Predict a Future Decision

While the high-rate/low-rate populations were split rather evenly (98 low-rate neurons, 89 high-rate neurons), a larger number of low-rate neurons might have been required to predict current and future trajectories. However, the total sample size that would be required to achieve above chance accuracy would likely vary depending on the algorithm’s inputs. Therefore, we reasoned that modeling future classification accuracies, under increasing sample sizes, would provide an approximate estimate for the required low-rate sample sizes to predict current and future trajectories.

Ito et al. (2015, 2018) demonstrated that population decoding is highly sensitive to the number of neurons included. Additionally, their data suggest a non-linear relationship between classification accuracy and neuron count. Thus, while preliminary analyses on our dataset revealed a positive correlation between classification accuracy and the number of neurons included from a given session (see “Materials and Methods” section), we wanted to start with a better characterization of the relationship between sample size and classifier accuracy.

**Figure 7:**
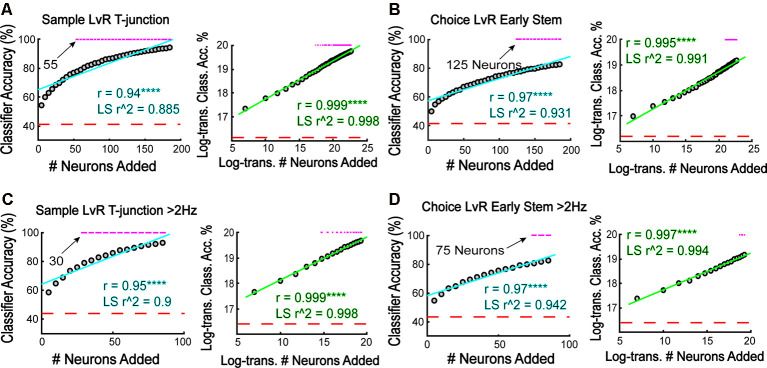
More neurons are required to predict a future choice than are required to reflect a forced behavior. **(A)** The classifier was iteratively trained/tested to predict whether rats were on the left or right trajectory at sample phase T-junction. Notice that 55 neurons were required to achieve above-chance prediction. Additionally, there is a strong positive correlation between classifier accuracy and the number of added neurons (*r* = 0.94, ****p = 3.2e-18), with the relationship being linear following log-transformation (*r* = 0.999, ****p = 4.3e-47). Linear regression modeling revealed lower residual error and greater prediction with log-transformed data (non-transformed data SSE = 448.2, *r*^2^ = 0.885; log-transformed data SSE = 0.03 *r*^2^ = 0.997). Each gray dot indicates the grand mean classification average over 5,000 shuffles of *N* (sample size) neurons. **(B)** The classifier was trained to predict choice phase left vs. right during early stem runs (second stem bin). Notice that 125 neurons were required to approach above chance accuracy. Additionally, the relationship between classifier accuracy and the number of neurons added was linear following a log-transformation (*r* = 0.97, ****p = 3.3e-22 not-transformed data; *r* = 0.995, ****p = 3.6e-37 log-transformed data; Pearson’s correlation). Linear regression modeling revealed lower residual error and greater prediction with log-transformed data (non-transformed SSE = 177, *r*^2^ = 0.931; log-transformed SSE = 0.102, *r*^2^ = 0.991). **(C)** High-rate neurons were trained to predict left vs. right trajectory at sample phase T-junction similar to **(A,B)**. Log-transformed data have higher linear correlation (non-transformed *r* = 0.953, ****p = 3.4e-09; log-transformed data *r* = 0.999, ****p = 2.1e-21). A linear regression revealed that log-transformed data have lower residual error and greater predictability (non-transformed data SSE = 153.2, *r*^2^ = 0.9; log-transformed data SSE = 0.012, *r*^2^ = 0.998). **(D)** High-rate neurons were trained to predict left vs. right trajectory during choice phase early stem traversal (second stem bin) with increasing number of added neurons to the classifier. Log-transformed data have a higher linear correlation (non-transformed *r* = 0.972, ****p = 7.1e-11; Log-transformed *r* = 0.997, ****p = 4.6e-18) exhibit lower residual error (non-transformed SSE = 59.2; log-transformed SSE = 0.03), and better model classification accuracy (non-transformed *r*^2^ = 0.942; log-transformed *r*^2^ = 0.994) using a linear regression. For all data in this figure, a *z*-test was used to determine significance and a magenta line indicates *p* < 0.05.

To estimate the relationship between the number of neurons and model performance, we iteratively and randomly added neurons to the classifier in increasing increments of five, training and testing the classifier 5,000 different times per sample size (we increased the number of iterations from 1,000 because we introduced more variability by selecting random neurons for classification). First, we focused on trajectory coding at T-junction from sample phase traversals given the high-accuracy noted in Figure 3A. Given the non-linear shape of the raw accuracy scores when plotted against sample size (Figure 7, left panels), we log-transformed the data (see “Materials and Methods” section), finding a stronger linear correlation (Figure 7A, right panel) when compared to the non-log-transformed data (Figure 7A, left panel). Additionally, we used sample size to predict classification accuracy with linear regression and found that log-transforming the dataset provided the lowest residual error (as assessed by the sum of squared errors), and allowed for a strong linear relationship between sample size and model performance (as assessed by an adjusted r-squared value).

We then used the same procedure to quantify the relationship between sample size and classifier accuracy using data taken from Figure 3D (trajectory coding on early stem traversals of choice phases) and Figure 5 (high-rate populations). In all cases examined (see Figures 7B through 7D), log-transforming the data provided the highest linear correlations and best-fit regression models. Therefore, there is a logarithmic relationship between the number of added neurons and the ability for the classifier to represent both current and future behaviors.

After plotting the number of neurons necessary to reach significance, we found that the required sample size to predict trajectory seemed to scale with cognitive demand. More specifically, at T-junction during the sample phase, around 55 neurons were required to predict left and right trajectories (Figure 7A). This is because 55 neurons provided the first classification accuracy above a chance level distribution. This is contrary to the 125 neurons required to predict a future decision (Figure 7B). Thus choice phase prediction during early stem running required a 56% increase in population size when compared to a forced turn. Additionally, when focusing on the high-rate population (Figures 7C,D), we found that 30 high-rate neurons were required to represent the current trajectory at T-junction (Figure 7C, left panel), while 75 neurons were required to reflect the future trajectory on choice phase (Figure 7D, left panel). Again, this demonstrates a relationship between sample size and cognitive demand but also reveals that a fewer number of high-rate neurons were required to reflect current and future behaviors in comparison to the entire population. However, looking back through the data, we noticed that the classifier performed around 84% accuracy when predicting if rats were on a sample right vs. choice left at T-junction (Figure 2G). Therefore, we iteratively trained/tested the classifier with increasing sample size, and found that the classifier required approximately 75 neurons to predict those conditions. Given that this sample size is 40% less than what is required to reflect a future decision, the lower accuracy observed between the sample right vs. choice left predictions do not confound an interpretation that cognitive demand and ensemble size may be linked.

Taken together, log-transforming the classification accuracy and sample size vectors, provides a backbone for modeling future accuracies given the tight relationship observed between the variables. Additionally, while we set out to characterize how model performance varied with the number of added neurons, we discovered a relationship between sample size and cognitive demand. Specifically, we found that a higher proportion of neurons are required to reflect decision-making processes when compared to a forced behavior that always amounts to a reward.

### Modeling Classifier Performance Using Linear Regression

**Figure 8:**
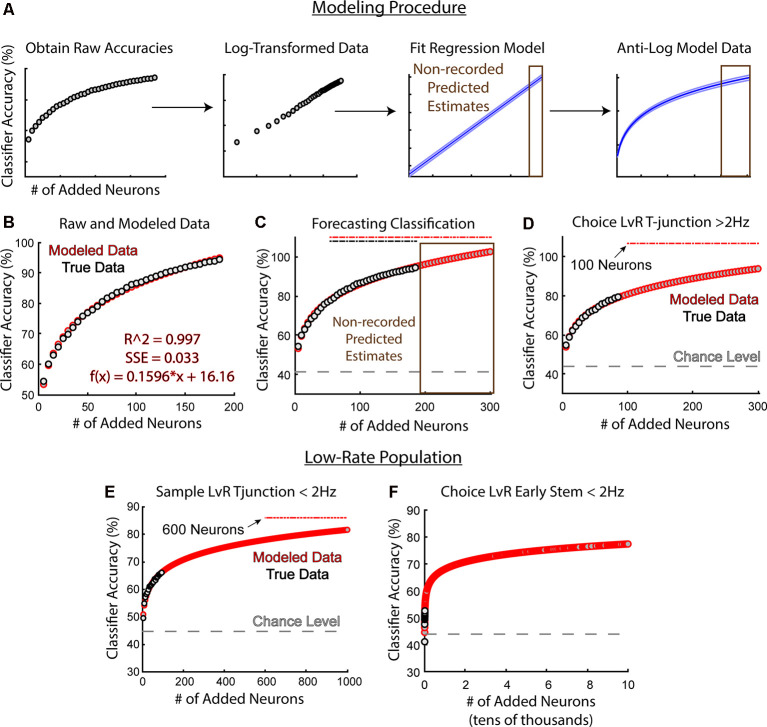
Modeling future classification accuracies reveal that low-rate neurons may weakly dissociate distinct trajectories at T-junction. **(A)** Classification accuracies and their corresponding sample-size (number of added neurons) were log-transformed, fit with a linear regression model, then converted back to classification accuracies *via* anti-log. Once the linear regression equation was obtained, we could forecast future classification accuracies (brown). Data were taken from the classification of trajectory at sample phase T-junction. **(B)** An example whereby the modeled estimates fit the recorded data with high accuracy (adjusted *R*^2^ = 0.997, SSE = 0.033) along with the regression equation. Data were taken from the classification of trajectory at sample phase T-junction. Gray dots indicate the averaged classification per sample size (averaged over 5,000 random combinations of neurons), while red dots indicate modeled data. **(C)** A demonstration of modeled accuracies from a population size that was not obtained. Data is the same as **(A)** and **(B)**. Recorded population *N* = 187. Gray dashed line indicates chance level accuracy. Brown box indicates predicted data. Dashed lines above model accuracies indicate significance utilizing a *z*-test. **(D)** Notice that a sample size of 100 neurons was predicted to yield a *p* < 0.05 (*Z* = 1.98, *p* = 0.047). The high-rate population did not exhibit the above-chance classification performance at T-junction (Figure 5C) due to the low sample size (actual sample size = 89 neurons). Compare this to Figure 7D; notice that 25% fewer neurons were required to predict a future decision. **(E)** A sample size of 600 neurons was estimated to predict trajectory at the T-junction of sample phases when considering a low-rate population (*Z* = 1.96, *p* = 0.0499 at 600 neurons). **(F)** Low-rate neurons do not effectively reflect future decision-making. Note the scale shown here is from 0 to 100,000 neurons and the data was taken from the second stem bin.

Given the strong linear relationship between sample size and model performance after log-transformations, we reasoned that we could take advantage of the linear regression model to predict future accuracies. Therefore, we created a simple modeling procedure that utilizes log-transformations (Figure 8A). This modeling procedure required us to first add neurons to the classifier iteratively and randomly (added in increasing increments of five, 5,000 different times per increment). We then log-transformed the data, fit a linear regression model to the dataset, then re-converted the modeled data to percent accuracy *via* anti-log. This procedure allowed us to use our output model equation to predict accuracies on sample sizes larger than our existing data set (Figure 8A). To further illustrate this process, we used classification data trained to predict trajectory at sample phase T-junction (Figures 8A,B). We were able to use the equation from the fit model (Figure 8B) to forecast future accuracies on larger sample-sizes (Figure 8C actual *N* = 187, predicted *N* = 300). To further assess the effectiveness of this procedure, we used data from Figure 5C, whereby the high-rate group fell short of predicting left/right trajectory at T-junction (trending effect: *Z* = 1.9, *p* = 0.053). We reasoned that this trending effect was due to insufficient sample size, and indeed found that 100 neurons were required to achieve above-chance accuracy, as opposed to the 89 high-rate neurons recorded (Figure 8D). However, it should be noted that rat 17–77, but not rat 18–28’s population, predicted choice phase trajectory at T-junction (Figure 4B). Additionally, removal of 17–77’s neuronal data precluded the ability for the classifier to perform above the chance level at T-junction (data not shown). While 17–77 did spend significantly more time sampling the T-junction on left choice phase runs when compared to right choice phase runs, the same was true for 18–28 (data not shown). Therefore, time-spent sampling one of the trajectories could not explain the ability for the classifier to dissociate trajectory at this location for 17–77. Given that approximately 100 high-rate neurons were required to predict T-junction location (Figure 8D), and because removal of low-rate neurons reduced classification accuracy at T-junction (compare Figure 3D to Figure 5C), we reason that classification prediction of this location requires a large ensemble of neurons.

Finally, once we developed the modeling procedure, we could examine whether low-rate neurons would predict current and future trajectories under larger sample sizes. Therefore, we first modeled future decoding accuracies of trajectory at sample phase T-junction using the low-rate group. Our procedure forecasted that approximately 600 neurons would be required to reflect the current trajectory at T-junction (Figure 8E). Additionally, 600 neurons would only predict the current trajectory at around 78% accuracy, with 1,000 neurons predicting the current trajectory at 82% accuracy. This is in contrast to the high-rate group, where only 30 neurons (5% of what would be required among the low-rate group) predicted sample phase trajectory at a level of 79% and 85 neurons predicted trajectory at 93%. These findings suggest that the low-rate population contains some level of trajectory dependent information during forced turns that always amount to a reward.

Next, we wondered if low-rate neurons contained information regarding a future trajectory. Based on Grosmark and Buzsáki (2016), we predicted that a low-rate group (after the well-learned performance of the DNMP task) would not be informative about future decisions. We originally modeled the classification accuracies to 1,000 neurons, like in Figure 8E, however, the accuracy was extremely low (60%). Thus, we modeled the data on 100,000 neurons and found a classification nearing 77%, still below significance (Figure 8F). We then attempted 200,000 neurons, but still, no significance was obtained (data not shown). Together, these findings support low-rate groups being weakly sensitive to forced behaviors, but likely not linked to future outcomes. However, it should be noted that drawing from classification accuracies near 50% could generate similar findings irrespective of the population. Additionally, modeling future classification accuracies on the raw classification outputs from the low-rate population in Figure 8F did not fit the data as robustly as our other analyses in Figure 8. Nonetheless, log-transforming the low-rate data provided the strongest correlation between classifier performance and sample size vectors (*r* = 0.86, *p* = 1.92e-06 Pearson’s correlation between log-transformed accuracy and sample size; *r* = 0.71, *p* = 0.007 Pearson’s correlation between classifier accuracy and sample size), while also providing the best fit model (adjusted *R*^2^ = 0.73, SSE = 0.232 linear regression between log-transformed classifier performance and sample size vectors; adjusted *R*^2^ = 0.48, SSE = 52.1 linear regression between classifier performance and sample size). Therefore, to the best of our knowledge and understanding, this modeling procedure is a leading approach to estimate future outcomes on the low-rate population.

Taken together, given that no increases in mean accuracy were noted among the low-rate population that would predict a future decision (Figure 5C), and given that our modeling procedures did not reveal strong evidence for the low-rate population predicting a future decision in the same stem-bin as the high-rate group (Figure 8F), our results support high-rate populations, but not low-rate populations, being relevant for decision-making.

## Discussion

In this study, we aimed to better characterize prefrontal representations of memory on a task that experimentally isolates encoding dominant and retrieval dominant phases of SWM. We demonstrate that high-firing rate, but not low-firing rate populations, effectively represent whether rats are actively engaged in a trajectory-dependent action during memory encoding, and also predict a future decision. Using a simple modeling procedure, we further reveal that the ability to reflect current trajectory dependent behaviors is weakly represented among low-rate populations. In essence, the ability to reflect trajectory-dependent behaviors seems to be a conserved phenomenon across the mPFC. This conclusion is also supported at the single-unit level, given that a similar proportion of high and low-rate neurons dissociate left and right turns at the T-junction. We suspect that through learning, certain populations are selected to collectively reflect current actions and predict future decisions. However, while differences in scaling could not explain our results, it is still possible that highly active neurons conferred a statistical advantage over the low active neurons, and that this statistical advantage may be independent of a special role that the high-rate neurons play in cognition. Nonetheless, focusing on the high-rate population, we demonstrated that a larger proportion of neurons were required to represent decision-making processes when compared to a forced behavior. This suggests that high-stakes decision-making processes likely employ a larger population than what is required to reflect a simple behavior that always amounts to a reward (i.e., a forced turn). Against our predictions, we found that apparent task phase coding was better explained by representations of overt trajectory-behaviors. Taken together, our study provides evidence that highly active mPFC ensembles are tuned to efficiently represent current and planned trajectory dependent actions, and that decision-making processes require larger ensemble sizes than forced behaviors in the rat.

It has been proposed that the mPFC acts as a counterpart to the motor cortex, whereby internalized action-plans are sent to limbic regions, as opposed to the spinal-cord for down-stream action (Buzsaki, 2019). Given that trajectory-dependent behaviors were almost always observed with above-chance decoding, and given that the population could predict a future decision in the absence of changes in behavior, our results may support such a role. However, we are not the first to provide evidence for a link between mPFC neuronal activity and trajectory-dependent behaviors. Euston and McNaughton (2006) demonstrated that neuronal activity from a sequence-task was strongly influenced by trajectory-dependent behaviors. Furthermore, Ito et al. (2015) used an open-field task and continuous alternation paradigm to demonstrate that mPFC neuronal activity was tightly linked to the movement direction, with spiking both preceding and following the motion. In conjunction with our work, it seems clear that the mPFC is strongly linked to the planning of trajectory-related actions, with goal-locations likely tuning this ability (Spellman et al., 2015). While these results are not the first to reveal evidence for the planning of a future decision (Baeg et al., 2003; Ito et al., 2015; Guise and Shapiro, 2017; Myroshnychenko et al., 2017), our study differs from past work that used a spatial alternation task with each traversal including encoding and retrieval dominant processes (Baeg et al., 2003; Ito et al., 2015). Additionally, our results differ from Myroshnychenko et al. (2017) in that we focused on trajectory-specific outcomes, as opposed to encoding/retrieval dominant phases of a task. Collectively, our findings support a highly specific role for the mPFC in the planning of future decisions, as mPFC lesions impair decision-making when prospective coding is thought to be required (Kesner, 1989). Future work should be aimed at using high-density recordings with real-time detection of neuronal patterns, so that inactivation procedures can shed light on the causal mechanisms of prefrontal representations.

Grosmark and Buzsáki (2016) reported that hippocampal neurons initially classified by a low firing rate profile acquire task-relevant activity and exhibited evidence of plasticity through learning. However, in our study, we find that highly active mPFC populations specifically support the encoding of on-going behavior and future trajectories during well-learned SWM behaviors. We suspect that these highly active mPFC neurons acquired rate-dependent increases through learning. If low-rate populations become tuned to reflect task-demands, one would expect to observe weak representations of trajectory-dependent behaviors and little evidence for prospective coding. Indeed, our modeling procedures suggest a weak representation of the current position during a forced behavior, with little evidence for the planning of a future outcome. On the contrary, when we focused on the high-rate group, we show that a comparably small sample size of high-rate neurons was required for the classifier to predict a trajectory in the presence of changes in behavior, as opposed to decision-making processes requiring on the order of 2.5–3.4 times the number of neurons. These findings allow us to conclude and predict the following: (1) mPFC populations are broadly tuned to reflect discrepancies in on-going behavior, given that both low-rate and high-rate neurons can collectively reflect trajectory in the presence of divergence in behavior; (2) highly active mPFC ensembles may be necessary to represent prospective experiences; and (3) there is a relationship between cognitive demand and the employed ensemble size in the rat mPFC. It should be noted that an alternative explanation is that highly active neurons confer a statistical advantage over low-active neurons in classifier outcomes. However, neurons classified by a session averaged firing rate of <2 Hz, can exceed 2 Hz at various maze locations (see Figure 6B). Additionally, analyses at the single-unit level revealed that a higher proportion of high-rate neurons were significantly modulated by early stem running, during choice phase traversals (see Figure 6D). It should also be noted that our results do not preclude a specific involvement of low-rate neurons in SWM behaviors. Specifically, low-rate neurons may be involved in temporal coding, as has been demonstrated in dCA1 neurons (Buzsaki, 2019). Moreover, it is reasonable to suspect that low-rate neurons provide support for certain population representations. Our data could support this conclusion for two reasons: (1) there were increases in the ability to predict trajectory among the low-rate population around T-junction (Figures 5B,C); and (2) there are trajectory modulated units during the choice phase (Figure 6C). Therefore, future studies are required to reveal the causal nature of high and low-rate populations in decision-making.

How do these results fit with and modify our current understanding of the role that the mPFC plays during SWM-guided behaviors at the systems level? It is well documented that the mPFC and hippocampus (HPC) are dually required for accurate SWM performance (Floresco et al., 1997; Churchwell et al., 2010; Churchwell and Kesner, 2011; Maharjan et al., 2018). Recently, work has demonstrated that the nucleus reuniens (Re) of the ventral midline thalamus coordinates mPFC-HPC communication (Ito et al., 2015; Hallock et al., 2016), explaining its importance for SWM usage (Hallock et al., 2013c, 2016; Layfield et al., 2015; Maisson et al., 2018). In line with these findings, Ito et al. (2015) demonstrated that the Re supports the ability for the dCA1 to reflect the future, but not past, trajectories. They provide further analyses to suggest that the mPFC likely informs the Re of the future trajectory, revealing a unidirectional path for the representations of prospective experiences. Thus, our work modifies the current understanding of how this circuit is implicated in memory-guided behavior by revealing that high-rate, but not low-rate, prefrontal ensembles likely inform dCA1 of plans. Additionally, our work suggests a relationship between ensemble size and cognitive demand, such that a greater number of neurons are required to inform down-stream targets of future actions. Finally, if mPFC coding for future trajectories is causal to the choice-action, we hypothesize that Re suppression during memory encoding disrupts future decision making (Maisson et al., 2018) by degrading prospective coding in the mPFC during the choice phase. Future work will target multisite recordings with optogenetic procedures to understand the impact of Re suppression on representations of SWM features among mPFC and HPC ensembles.

In summary, our study experimentally dissociated encoding dominant and retrieval dominant phases of SWM to better characterize prefrontal population representations. We show that high-firing but not low-firing mPFC populations, efficiently represent current and future trajectory-dependent actions. We also provide evidence of a relationship between prefrontal ensemble size and cognitive demand, while demonstrating that apparent task phase coding is explained by trajectory-dependent behaviors. Together, our findings extend on our understanding of the functional role the mPFC plays during SWM guided behavior, putting a specific emphasis on the representations of on-going behavior and future actions.

## Data Availability Statement

The raw data supporting the conclusions of this article will be made available by the authors, without undue reservation.

## Ethics Statement

The animal study was reviewed and approved by University of Delaware Institutional Animal Care and Use Committee (IACUC).

## Author Contributions

JS and AG designed the experiment and wrote the manuscript. JS recorded and analyzed the data.

## Conflict of Interest

The authors declare that the research was conducted in the absence of any commercial or financial relationships that could be construed as a potential conflict of interest.

## Footnotes

^1^http://www.github.com/GriffinLabCode/GriffinCode
